# Exposure to particulate pollutant increases the risk of hospitalizations for Sjögren’s syndrome

**DOI:** 10.3389/fimmu.2022.1059981

**Published:** 2022-12-15

**Authors:** Tian-Ping Zhang, Jing Dou, Li Wang, Shan Wang, Ping Wang, Xiao-Hui Zhou, Chun-Mei Yang, Xiao-Mei Li

**Affiliations:** ^1^ Department of Rheumatology and Immunology, The First Affiliated Hospital of USTC, Division of Life Sciences and Medicine, University of Science and Technology of China, Hefei, China; ^2^ Bengbu Medical College, Bengbu, China; ^3^ Department of Rheumatology, The First People’s Hospital of Hefei (Binhu Hospital), Hefei, China; ^4^ Department of Rheumatology, The First People’s Hospital of Hefei, Hefei, China; ^5^ Department of Rheumatology and Immunology, The Third People’s Hospital of Hefei, Hefei, China; ^6^ Department of Scientific Research, The First Affiliated Hospital of USTC, Division of Life Sciences and Medicine, University of Science and Technology of China, Hefei, China

**Keywords:** particulate pollutant, Sjögren’s syndrome, autoimmune diseases, time-series, PM2.5, PM10

## Abstract

**Objective:**

Numerous researches have reported the role of air pollution in the development of autoimmune diseases. However, few have evaluated the relationship between inhalable particulate matter (PM) exposure and Sjögren’s syndrome (SS). This study aimed to analyze the association between exposure to two particulate pollutants (PM_2.5_, PM_10_) and SS-related hospitalizations.

**Methods:**

Daily data were obtained on PM_2.5_ and PM_10_, meteorological factors, and hospital hospitalizations for SS between 2016 and 2021. The daily data on PM_2.5_ and PM_10_, meteorological factors, and the number of SS hospitalizations were collected between 2016 and 2021. A distributed lag non-linear model and a generalized linear model were established to explore the association between PM_2.5_ and PM_10_ exposure and hospitalizations for SS. Stratified analyses were performed to explore possible gender-, age-, and season-related differences in PM_2.5_ and PM_10_ effects.

**Results:**

Exposure to PM_2.5_ was related to the evaluated risk of hospitalizations for SS (*RR*=1.015, 95% *CI*: 1.001-1.029, lag 3 day), similarly, PM_10_ exposure had a statistically significant positive association with SS hospitalizations (*RR* =1.013, 95% *CI*: 1.001-1.026, lag 3 day). Stratified analyses found that exposure to PM_2.5_ and PM_10_ exhibited higher impact on SS-related hospitalizations in female patients and exposure to PM_2.5_ was also associated with the higher risk of SS-related hospitalizations in patients aged ≥ 65 years. In addition, exposure to PM_2.5_, PM_10_ in colder season were more likely to increase SS-related hospitalizations.

**Conclusion:**

Our findings suggested that exposure to PM_2.5_ and PM_10_ were significantly linked to an elevated risk of hospitalizations for SS.

## 1 Introduction

As a common, systemic autoimmune disease, Sjögren’s syndrome (SS) is characterized by lymphocyte proliferation and progressive destruction of the exocrine glands. The major clinical manifestations of these patients include dryness of the mouth and eyes, joint pain, and fatigue, which seriously affect the life quality of SS patients (1). Moreover, these patients also exhibit other clinical manifestations involving multiple systems, such as the nervous system, musculoskeletal system, kidney, blood vessels, skin, and lung (2). However, the potential pathogenesis of SS has not been fully elucidated. Some studies have shown that host genetics and external environmental factors could regulate the pathogenesis of SS through various mechanisms. Genetic predisposition accounts for approximately 30% of autoimmune diseases; environmental factors including air pollution are also involved the development of these diseases (3, 4). Ambient air pollution levels have been reported to have acute adverse effects on population health, primarily in terms of respiratory and cardiovascular conditions (5, 6); their role in autoimmune diseases has gained attention in recent years (7). Environmental exposure to air pollution may mediate the development of autoimmune diseases by increasing inflammatory responses, oxidative stress, and immune responses. In this context, a previous study demonstrated that nitrogen dioxide (NO_2_) and sulfur dioxide exposure might increase the number of first admissions for systemic lupus erythematosus (8). Another study reported significative association between short-term exposure to NO_2_, carbon monoxide and hospitalizations for acute gout (9).

As common air pollutants, particulate matter (PM) pose an important threat to human health; the impact of PM_2.5_ (aerodynamic diameter ≤ 2.5 μm) and PM_10_ (aerodynamic diameter ≤ 10 μm) have been studied more extensively in some diseases. Studies increasingly suggest that PM_2.5_ exposure is related to many inflammatory dermatoses, including atopic dermatitis, acne vulgaris, and psoriasis (10–12). PM_2.5_ exposure has also been found to cause local or systemic inflammatory responses and regulation of bodily immune functions, thereby leading to the development of autoimmune diseases (13). One birth cohort study found that PM_10_ exposure in the third trimester of pregnancy could increase the levels of interleukin-1β in cord blood (14); notably, overexpression of interleukin-1β had been found to lead to the development of autoimmune diseases (15). Although these findings suggest that PM exposure may be involved in the pathogenesis of autoimmune diseases, further studies are needed for investigation and validation.

Air pollution was found to be strongly related to the severity of eye irritation, ocular surface abnormalities in SS patients, and the findings also suggested that it could influence the risk of developing SS (16–18). However, available data on the relationship between PM exposure and SS remain scarce. In addition, the lag-response effect has not been considered in several previous time-series studies. This time-series study aimed to explore the potential correlation between PM_2.5_ and PM_10_ exposure and hospitalizations for SS in the city of Hefei; it also aimed to identify the susceptible population by gender, age, and the seasonal variability.

## 2 Subjects and methods

### 2.1 Study subjects

This time-series study analyzed the data on SS-related hospitalizations in Hefei (31°52′N, 117°17′E; the capital city of Anhui Province, China) between January 1, 2016 and December 31, 2021. The total covered area of this city is 11,445.1 km^2^, with a population of 9.465 million in 2021, and experiences a monsoon-influenced humid subtropical climate.

We collected the daily records of hospital admissions among patients with SS from three hospitals in Hefei (the First Affiliated Hospital of University of Science and Technology of China, the First People’s Hospital of Hefei, and the Third People’s Hospital of Hefei) during the study period. The diagnosis of all SS patients was made by rheumatologic clinicians according to the 2002 American European Consensus Group (AECG) classification criteria. Data pertaining to gender, residential address, and date of hospitalizations were also obtained. Patients with a residential address outside Hefei, incomplete demographic information, and a confirmed positive COVID-19 test were all excluded from the study. In addition, the patients with SS who were admitted to other departments (besides the Department of Rheumatology and Immunology) due to other reasons were also excluded. This study was approved by the Ethics Committee of the First Affiliated Hospital of University of Science and Technology of China.

### 2.2 Pollutants and meteorological data

The data pertaining to particulate matter (24-h data for PM_2.5_ and PM_10_) and other air pollutants (24-h for NO_2_) were collected from the Hefei Environmental Monitoring Center (originally collected from 10 air quality monitoring stations). The daily concentration of PM_2.5_, PM_10_ in Hefei was calculated by the average value of the 10 monitoring stations. This study also collected the meteorological data, including mean temperature (MT, °C) and relative humidity (RH, %), from the China Meteorological Data Service Center (http://data.cma.cn/) among the same period.

### 2.3 Statistical analysis

The influence of PM_2.5_, PM_10_ on SS-related hospitalizations was assessed using a distributed lag non-linear model combined with a generalized linear model. These two models were used to describe the additional lag-response correlation and traditional exposure-response correlation, respectively. As daily admission was considered a minor probability event in patients with SS, the distributed lag non-linear model with quasi-Poisson distribution was employed to analyze the correlation between particulate pollution and SS hospitalizations. Spearman analysis was used to analyze the correlation between each covariate, and two variables with correlation coefficient less than 0.7 could not be included in a same model at the same time to avoid multicollinearity.

The models finally used for PM_2.5_ and PM_10_ were as follows:


$$Y_{t}\sim quasipossion(\mu_{t})$$

$$Log{\left(\mu_{t1}\right)}_{PM2.5}=\alpha_{1}+\beta_{1}PM_{2.5t,l}+ns\left(NO_{2},3\right)+ns\left(MT,3\right)$$

$$+ns\left(RH,3\right)+ns\left(time,6*6\right)+\eta_{1}DOW_{t}+\gamma_{1}Holiday_{t}$$

$$Log{\left(\mu_{t2}\right)}_{PM10}=\alpha_{2}+\beta_{2}PM_{10t,l}+ns\left(NO_{2},4\right)+ns\left(MT,4\right)$$

$$+ns\left(RH,4\right)+ns\left(time,6*6\right)+\eta_{2}DOW_{t}+\gamma_{2}Holiday_{t}$$

The subscript *t* represented the day of observation, *α* referred to the intercept of every model and *Yt*, *μt* respectively were the actual, expected incidence of SS-related hospitalizations on day *t*. Take the PM_2.5_ Model as an example, *PM_2.5t,l_* represented the dlnm cross basis matrix of *PM_2.5t_*, *l* was the lag day, *β_1_* was set as the vector of *PM_2.5t_*, and ns() denoted the natural cubic spline function. A natural cubic spline curve of time with 6 dfs/year was used to adjust for seasonality and long-term trends (17). The dummy variable *DOW* and the two-category variable *Holiday_t_* were used to adjust the effect of weekends and public holidays, respectively. The optimal *dfs* and the final model parameters for particulate pollution were confirmed based on the values of the quasi Akaike Information Criterion.

We performed stratified analyses were to determine the susceptible population according to age (Age ≤ 40 years, 41 years ≤ Age ≤ 64 years, Age ≥ 5 years), gender (The male patients were too few and this study only analyzed the female SS patients), and season (hot season: April to September and cold season: October to March). This study adopted R 3.6.1 (http://www.R-project.org) with dlnm and splines packages to conduct statistical analyses and visualization, and *P<*0.05 (two-sided) was considered to be statistically significant.

## 3 Results

### 3.1 Basic analysis

**Figure 1:**
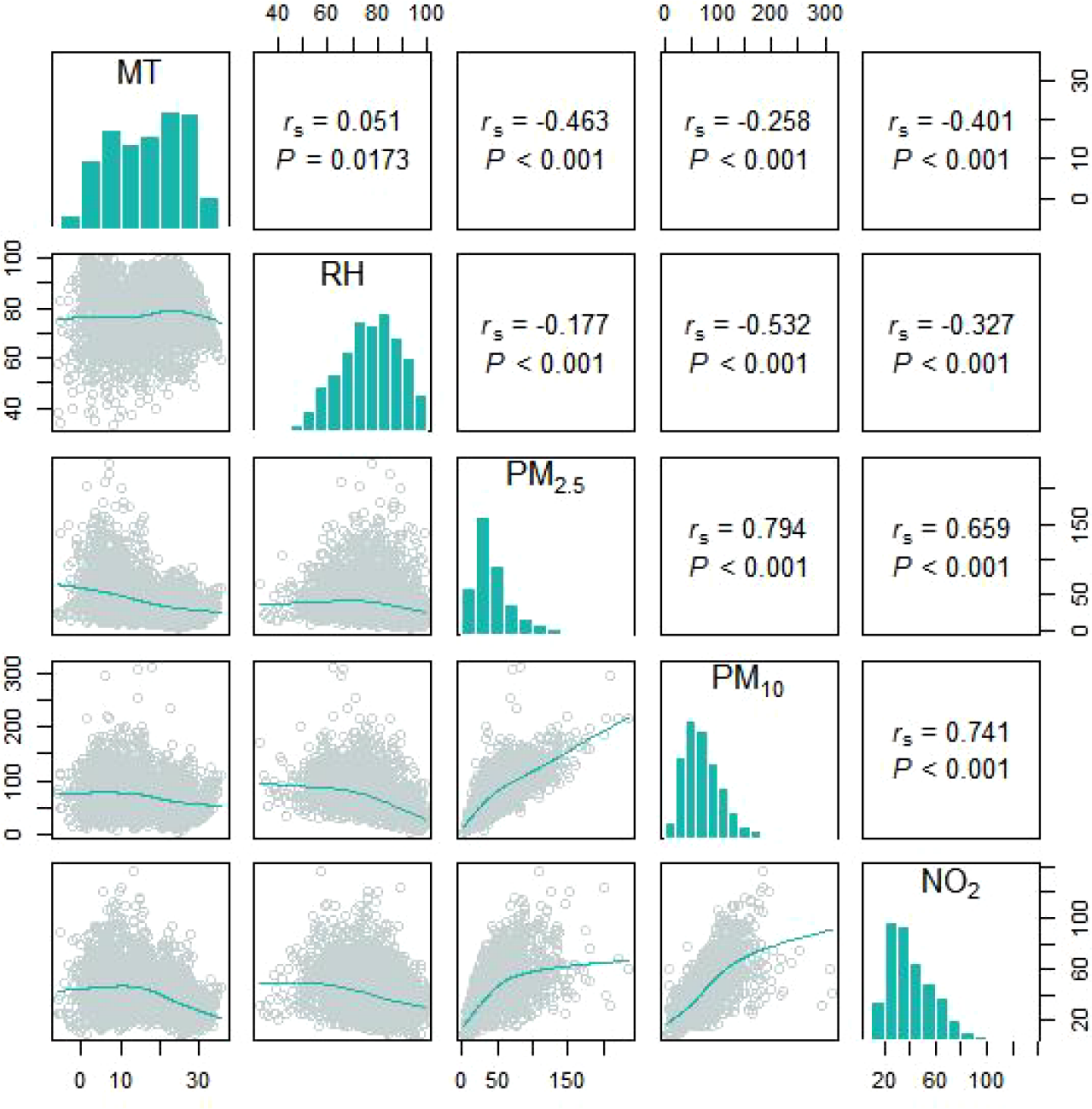
The spearman’s correlation coefficients between different meteorological factors and particulate pollutants.

**Figure 2:**
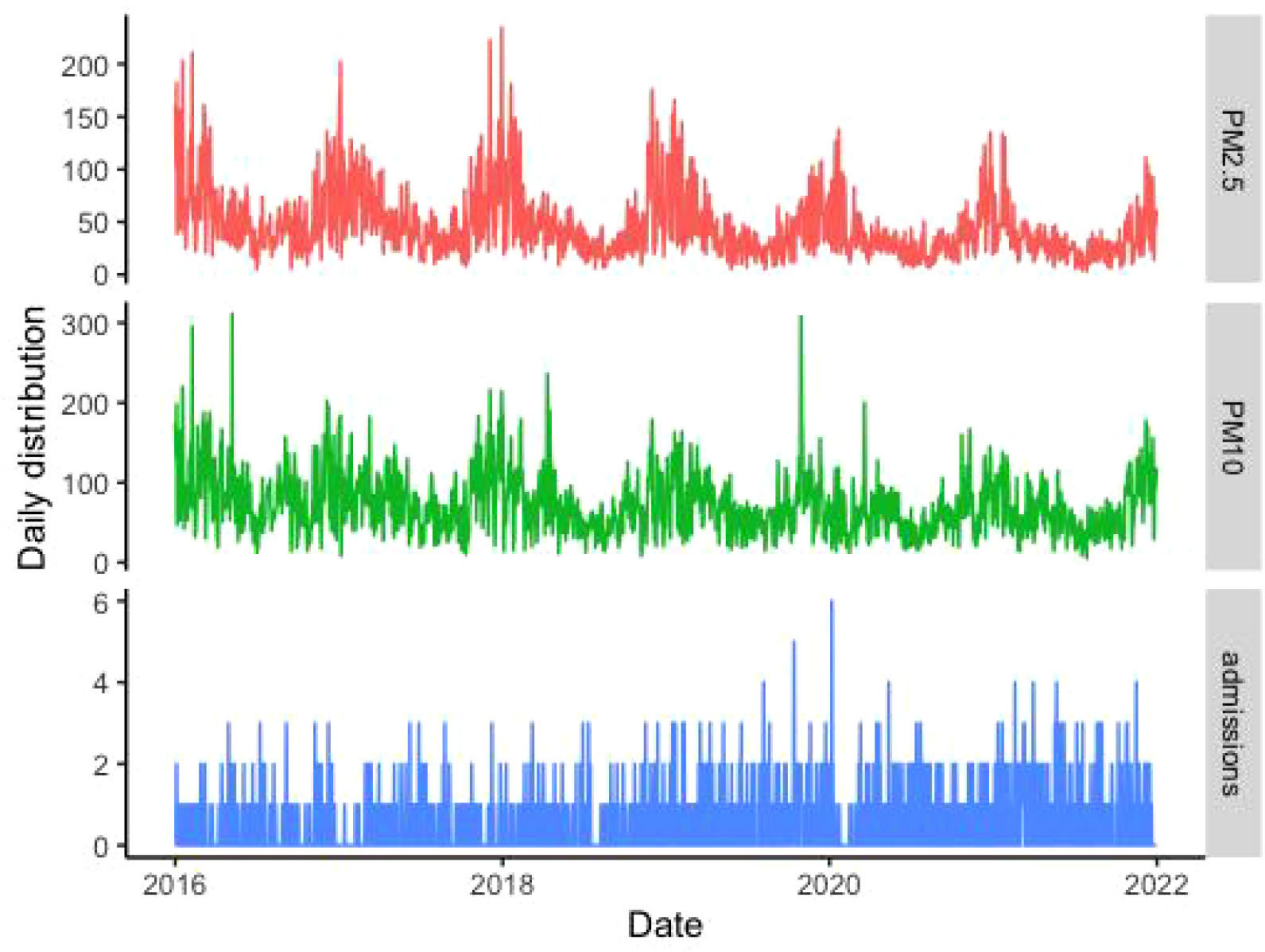
The time series of PM_2.5_, PM_10_, and SS hospitalizations in Hefei from 2016 to 2021.

The related information pertaining to SS-related hospitalizations, particulate matter pollution, and meteorological factors are presented in **Table 1**. Data were obtained for a total of 1119 SS-related hospitalizations between January 1, 2016 and December 31, 2021, and the daily number of SS hospitalizations ranged 0 to 6. Among the included patients, 1061 (94.8%) were male and 350 patients (31.3%) were aged ≥ 65 years. The average daily concentrations of PM_2.5_ and PM_10_ were 45.40 μg/m^3^ and 71.85 μg/m^3^, respectively. According to correlation analysis, the correlation coefficient between PM_2.5_ and PM_10,_ as well as PM_10_ and NO_2,_ was greater than 0.7 (**Figure 1**). The temporal trends of PM_2.5_, PM_10_, SS hospitalizations from 2016 to 2021 in Hefei were showed in **Figure 2**.

**Table 1:** The basic information of SS hospitalizations, meteorological data and particulate pollutants.

Variables	N	Mean (SD)	Min	*P_25_*	*P_50_*	*P_75_*	*P_90_*	Max
Admissions	1119	0.51(0.77)	0	0	0	1	2	6
Male	58	0.03(0.16)	0	0	0	0	0	1
Female	1061	0.48(0.75)	0	0	0	1	1	6
Age ≤ 40 years	159	0.07(0.27)	0	0	0	0	0	2
41 years ≤ Age ≤ 64 years	610	0.28(0.56)	0	0	0	0	1	4
Age ≥ 65 years	350	0.16(0.41)	0	0	0	0	1	4
Hot season	580	0.26(0.61)	0	0	0	0	1	4
Cold season	539	0.25(0.60)	0	0	0	0	1	6
MT, °C	–	16.77(9.26)	-5.9	8.7	17.2	24.725	28.3	35.6
RH, %	–	76.99(12.26)	33	69	78	86	93	99
PM_2.5_, μg/m^3^	–	45.40(30.05)	3	25	37	56.7	85	235
PM_10_, μg/m^3^	–	71.85(36.85)	5	45	66	93	119.7	311

### 3.2 Association between particulate pollutants and hospitalizations for SS

#### 3.2.1 Overall effect

**Figure 3:**
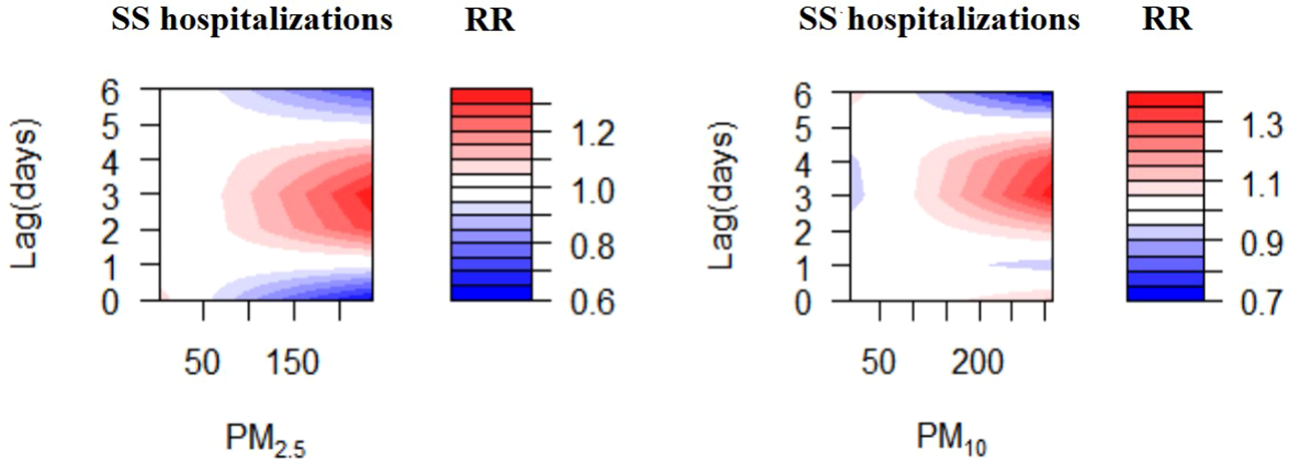
Contour plots for relative risk of hospitalizations for SS along PM2.5, PM10 at lag periods.

The exposure-response correlations between particulate pollutant (PM_2.5_ and PM_10_) exposure and SS hospitalizations on different lag days has been presented in **Figure 3**. Exposure to the high concentrations of PM_2.5_ and PM_10_ (reference concentrations of 37 μg/m^3^ and 66 μg/m^3^, respectively) were significantly related to the risk of SS-related hospitalizations. The concentration-response relationships between PM_2.5_ and PM_10_ concentrations and SS-related hospitalizations are presented in **Figure S1**.

#### 3.2.2 The effect of PM_2.5_ on SS hospitalizations

**Figure 4:**
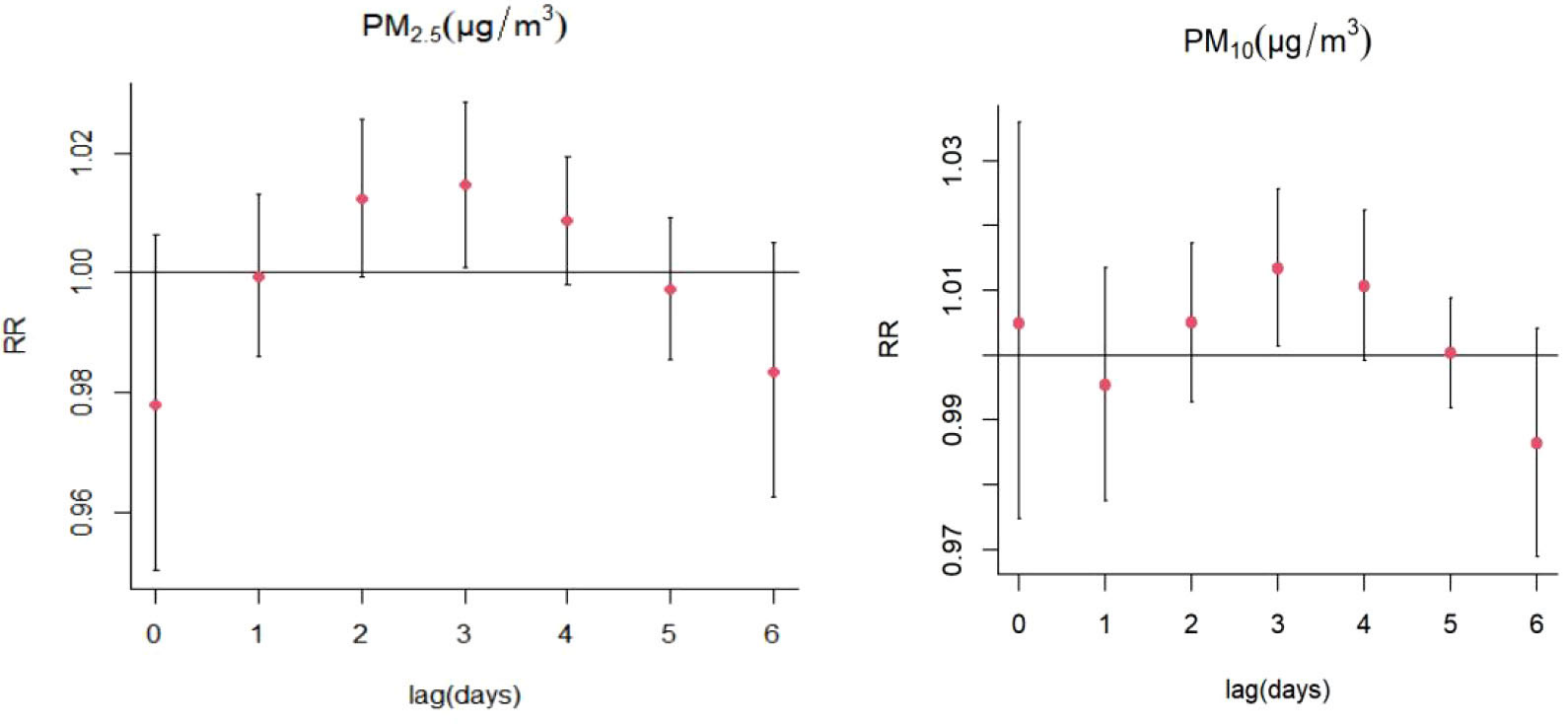
Lag-specific relative risks (%) in hospitalizations for SS per 10 unit increase in daily mean concentrations of PM_2.5_, PM_10_.

**Figure 5:**
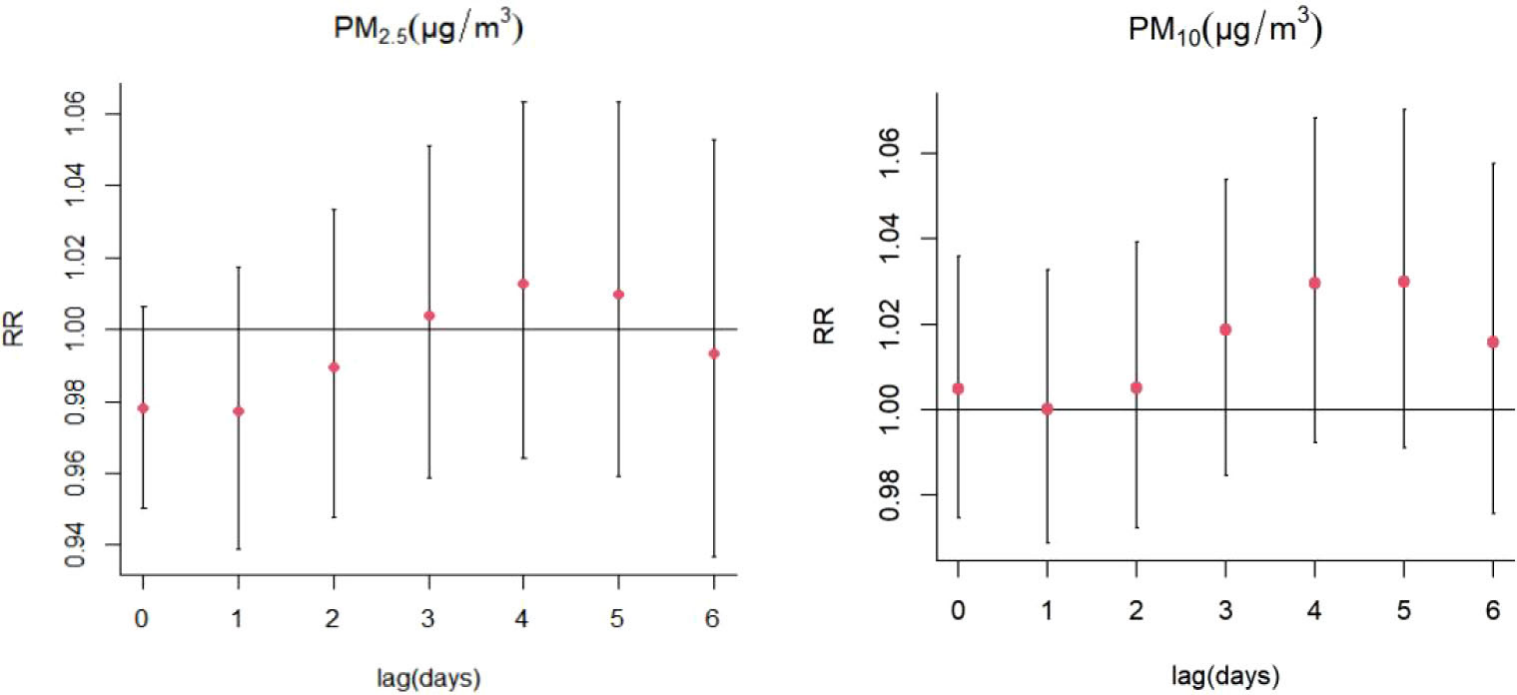
Cumulative risks (%) in hospitalizations for SS per 10 unit increase in daily mean concentrations of PM_2.5_, PM_10_.

**Figure 6:**
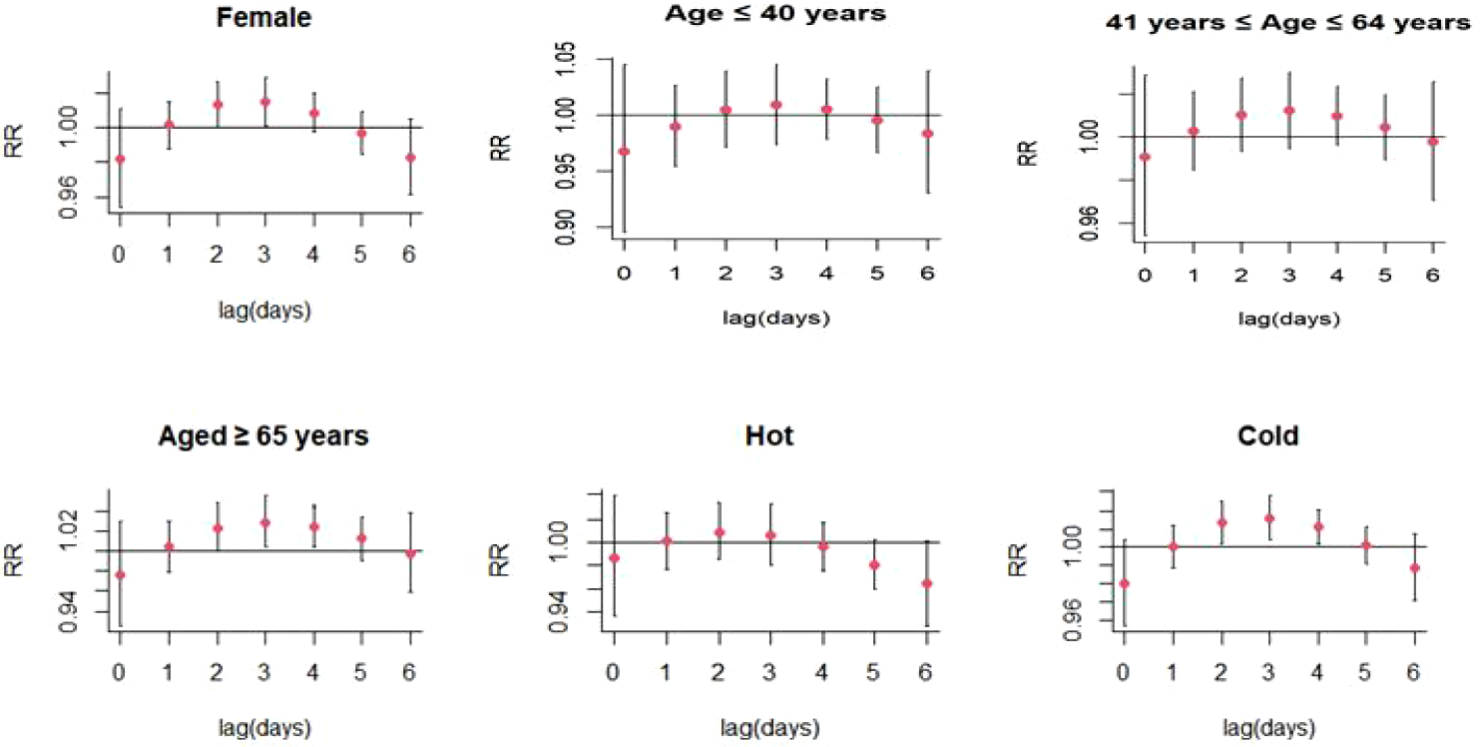
Lag-specific relative risks (95% CI) of SS hospitalizations per 10 unit increase in the daily concentrations of PM_2.5_ in model stratified by age, gender, and season.

Increases in PM_2.5_ concentrations by 10 μg/m^3^ were found to cause significant single-day effects on the association between exposure to PM_2.5_ and SS-related hospitalizations at lag 3 day (relative risk [RR]: 1.015, 95% confidence interval [CI]: 1.001-1.029) (**Figure 4**; **Table S1**); no cumulative effects were observed for PM_2.5_ (**Figure 5**). On stratified analyses, PM_2.5_ exposure remained positively associated with SS-related hospitalizations in female patients (lag 3 day, RR: 1.015, 95% CI: 1.001-1.029), patients aged ≥ 65 years (lag 3 day, RR: 1.029, 95% CI: 1.004-1.054), and colder months (lag 3 day, RR: 1.016, 95% CI: 1.005-1.028) (**Figure 6**; **Table S2**).

#### 3.2.3 The effect of PM_10_ on SS hospitalizations

**Figure 7:**
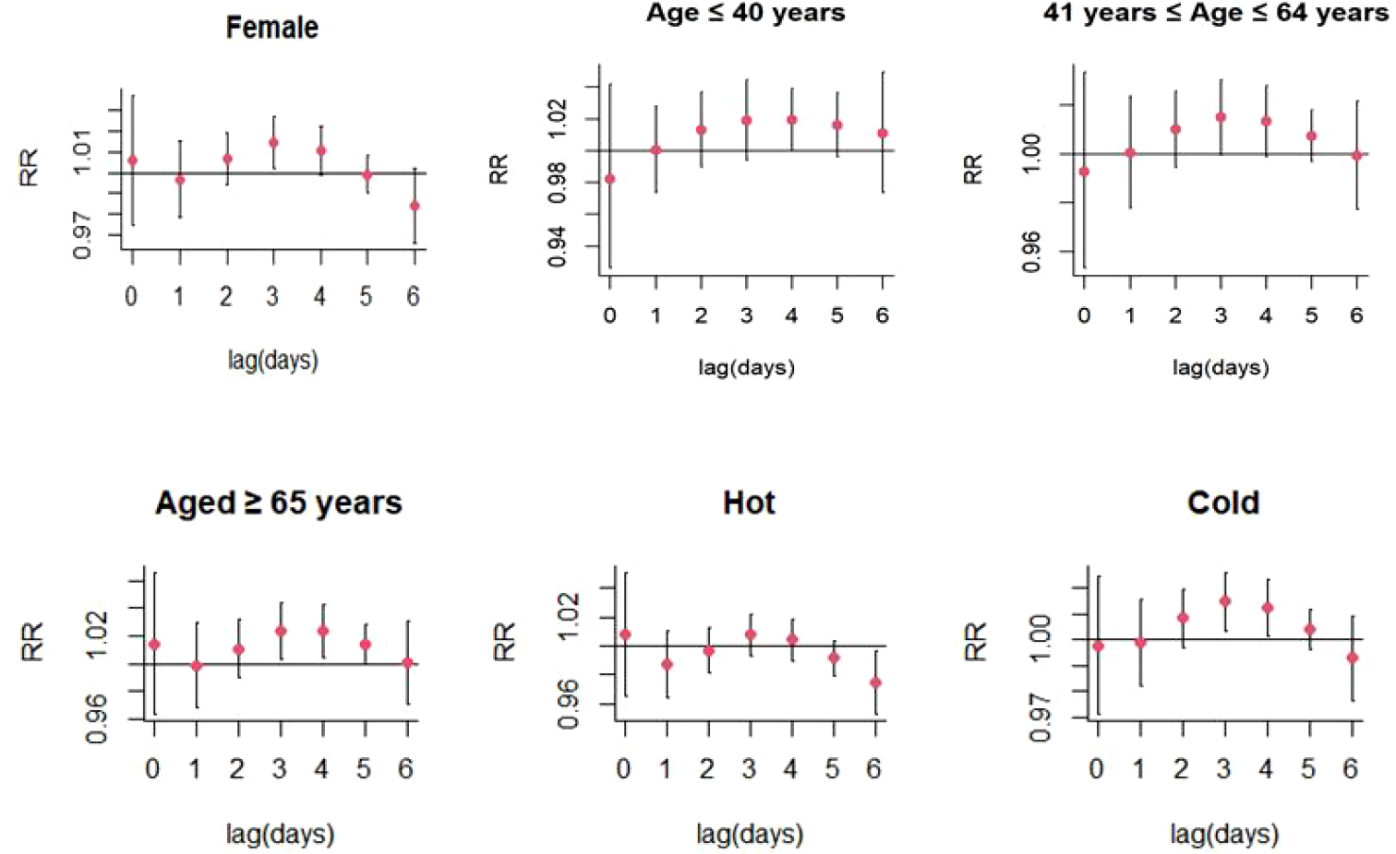
Lag-specific relative risks (95% CI) of SS hospitalizations per 10 unit increase in the daily concentrations of PM_10_ in model stratified by age, gender, and season.

For every 10 μg/m^3^ increase in PM_10_ concentrations, the risk of SS hospitalizations increased at lag 3 day (RR: 1.013, 95% CI: 1.001-1.026) (**Figure 4**; **Table S3**). Significant positive relationship were also existed between PM_10_ exposure and SS-related hospitalizations in female patients (RR: 1.014, 95% CI: 1.002-1.027; lag 3 day) and colder months (RR: 1.015, 95% CI: 1.004-1.026) (**Figure 7**; **Table S4**). The stratified analyses also suggested that PM_10_ exposure was both significantly associated with SS-related hospitalizations in three different age groups (Age ≤ 40 years: RR: 1.019, 95% CI: 1.001-1.039, lag 4 day; 41 years ≤ Age ≤ 64 years: RR: 1.015, 95% CI: 1.001-1.030; lag 3 day; Age ≥ 65 years: RR: 1.024, 95% CI: 1.003-1.044; lag 3 day) (**Figure 7**; **Table S4**).

### 3.3 Sensitivity analyses

We performed sensitivity analyses to verify the stability of the two models with the ns function after adjusting the *dfs* for air pollutants (3–5 *dfs*), meteorological factors (3–5 *dfs*), and time (6–8 *dfs* per year). For PM_2.5_ model, when the *dfs* for time was 6, we adjust the *dfs* for air pollution from 3 to 5 in turn (The *dfs* for air pollutants was the same as for meteorological factors), and get three figures (**Figure S2**). When the *dfs* for air pollutants and meteorological factors were 3, we adjusted the *dfs* for time from 6 to 8 in turn, and get three figures (**Figure S2**). For PM_10_ model, when the *dfs* for time was 6, we adjust the *dfs* for air pollution from 3 to 5 in turn (The *dfs* for air pollutants was the same as for meteorological factors), and get three figures (**Figure S3**). When the *dfs* for air pollutants and meteorological factors were 4, we adjusted the *dfs* for time from 6 to 8 in turn, and get three figures (**Figure S3**).

The results suggested that the primary results of these models were relatively stable with the adjustment of different *dfs*, therefore, these models established in our study could be considered reliable (**Figures S2, S3**).

## 4 Discussion

This time-series study assessed potential associations between exposure to the high concentrations of PM_2.5_, PM_10_ and the risk of SS-related hospitalizations. The findings revealed certain points of interest. First, PM_2.5_ and PM_10_ exposure was found to have a positive impact on SS-related hospitalizations in Hefei. Second, on stratified analyses by sex, age, and season, the effects of PM_2.5_ and PM_10_ on SS-related hospitalizations remained significant in female patients and cold season.

At present, some studies had evaluated the effect of PM_2.5_ and PM_10_ exposure on autoimmune disease-related hospitalizations. In their time-series study, Wu et al. showed a positive correlation between exposure to high concentrations of PM_2.5_ and the number of rheumatoid arthritis hospital readmissions (19). Another study suggested that exposure to PM_2.5_ played a significant effect on the risk of recurrence in systemic lupus erythematosus patients (8). However, another study suggested that PM_2.5_ exposure was not linked to the risk of gout-related hospitalizations (9). The findings from these studies suggested that the influence of particulate matter exposure on the risk of developing autoimmune diseases was not consistent; this might be attributed to the different modes of pathogenesis of autoimmune diseases. In the present study, we found that exposure to PM_2.5_ significantly increased the risk of SS-related hospitalizations; exposure to PM_10_ also had a similar effect. Consistent with one previous study, Chen et al. found that PM_2.5_ exposure was associated with increased risk of SS outpatient visits (20). These two studies provided strong evidence to confirm the involvement of PM_2.5_ in the development of SS. The effects of air pollutants on the development of autoimmune diseases may be attributed to several factors, including T-cell imbalance, proinflammatory cytokine expression, oxidative stress, and local lung inflammation (21). PM_2.5_ has been proven to be capable of activating nuclear factor kappa B, cyclooxygenase 2, and toll-like receptor 4 signaling pathways, thereby inducing inflammation in macrophages. PM_2.5_ contains high-affinity aryl hydrocarbon receptor ligands (22, 23); the particles could therefore enhance T helper 17 cell differentiation, promote regulatory T cell production, and modulate autoimmunity by targeting aryl hydrocarbon receptors (24, 25). Our study confirmed a meaningful association between PM exposure and SS hospitalizations and provided critical epidemiological evidence for investigation of the role of PM in autoimmune diseases.

Subgroup analyses were conducted according to different sex and age in order to identify vulnerable groups. Seasonal differences (hot season vs. cold season) in the effect of PM_2.5_ and PM_10_ exposure on SS-related hospitalizations were also investigated. The results of our analyses suggested that PM_2.5_ and PM_10_ exposure played significant impact on the risk of SS-related hospitalizations in female patients. Similar to the results of another research (26), the risk of hospitalizations in female patients was particularly influenced by extreme environmental factors such as extremely cold temperatures, extreme levels of humidity, and longer durations of sunlight. This might be attributed to gender-based differences in immune defenses, which could have in turn influenced the effect of PM exposure on the risk of SS-related hospitalizations. A previous study found the coefficient of variation of lipopolysaccharide-induced and lipoteichoic acid-induced cytokine responses to be higher in blood samples from females than from males; this finding was consistent for all parameters and stimuli measured (27). Because the incidence of SS in female was significantly higher than that in male, the number of male SS patients in this study was too few. Therefore, the effect of PM_2.5_ and PM_10_ on SS-related hospitalizations was not analyzed in male SS patients in this study.

Compared to younger patients, the older patients with SS were more susceptible to PM_2.5_ exposure in this study. This might be attributed to age-related differences in the development of immune responses, which decline in old age (28). It should be noted that the effect of PM_10_ exposure on SS-related hospitalizations was significant in three age groups (Age ≤ 40 years, 41 years ≤ Age ≤ 64 years, Age ≥ 65 years), suggesting that this effect might not be affected by age. The correlation between PM_2.5_ and PM_10_ exposure and increased risks of SS-related hospitalizations was stronger in the cold season; this might be explained by the low levels of humidity in these months. López-Miguel et al. suggested that exposure to dry conditions might deteriorate lacrimal gland function in patients with SS who had dry eye symptoms; this might be mediated by promotion of inflammatory activity (29).In this study, there were also several limitations. First, this study only collected some demographic data of SS patients, but did not include clinical manifestations, complications and other characteristics, so other stratified analyses could not be carried out. Second, the hospitalizations data for patients with SS were obtained from three hospitals in Hefei; however, it was not possible to include representative data for the entire population of Hefei. Third, as it had an ecological design, this study could not provide evidence for a causal relationship between PM_2.5_ and PM_10_ exposure and SS-related hospitalizations; this limitation was similar to that of certain previous time-series studies (9, 19). However, our study had certain strengths. This should be the first study to explore the association between exposure to PM_2.5_ and PM_10_ and SS-related hospitalizations in an area with a monsoon-influenced humid subtropical climate. This study also assessed the impact of gender age, and season on this association and identified the vulnerable population.

In summary, our results showed a strong association between PM_2.5_, PM_10_ exposure and the risk of SS-related hospitalizations. Moreover, female patients were more susceptible to PM_2.5_ and PM_10_ exposure, and the cold season increased the number of SS-related hospitalizations. This study provided valuable insights into the involvement of environmental factors in the pathogenesis of SS. As particulate pollutants remain a serious environmental issue, epidemiological studies based on larger sample sizes and functional studies are needed to investigate the impact of PM_2.5_, PM_10_, and other ultra-fine PM on SS.

## Data availability statement

The original contributions presented in the study are included in the article/**Supplementary Material**. Further inquiries can be directed to the corresponding authors.

## Ethics statement

This study was approved by the Ethical Committee of the First Affiliated Hospital of USTC (Hefei, Anhui, China). Written informed consent for participation was not required for this study in accordance with the national legislation and the institutional requirements.

## Author contributions

X-ML, C-MY, and T-PZ designed the study. LW, SW, PW, and X-HZ participated in the data collection. T-PZ and JD conducted the data analysis, T-PZ drafted the manuscript. X-ML and C-MY contributed to manuscript revision. All the authors approved the final submitted version.

## References

[1] Baldini C Talarico R Tzioufas AG Bombardieri S. Classification criteria for sjogren's syndrome: a critical review. J Autoimmun (2012) 39(1-2):9–14. doi: 10.1016/j.jaut.2011.12.006 22209352

[2] Qin B Wang J Yang Z Yang M Ma N Huang F. Epidemiology of primary sjögren's syndrome: a systematic review and meta-analysis. Ann Rheum Dis (2015) 74(11):1983–9. doi: 10.1136/annrheumdis-2014-205375 24938285

[3] Vojdani A Pollard KM Campbell AW. Environmental triggers and autoimmunity. Autoimmune Dis (2014) 2014:798029. doi: 10.1155/2014/798029 25610638 PMC4290643

[4] Rosenblum MD Remedios KA Abbas AK. Mechanisms of human autoimmunity. J Clin Invest (2015) 125(6):2228–33. doi: 10.1172/JCI78088 PMC4518692 25893595

[5] Basith S Manavalan B Shin TH Park CB Lee WS Kim J. The impact of fine particulate matter 2.5 on the cardiovascular system: A review of the invisible killer. Nanomaterials (Basel) (2022) 12(15):2656. doi: 10.3390/nano12152656 35957086 PMC9370264

[6] Huang K Ding K Yang XJ Hu CY Jiang W Hua XG. Association between short-term exposure to ambient air pollutants and the risk of tuberculosis outpatient visits: A time-series study in hefei, China. Environ Res (2020) 184:109343. doi: 10.1016/j.envres.2020.109343 32192989

[7] Celen H Dens AC Ronsmans S Michiels S De Langhe E. Airborne pollutants as potential triggers of systemic autoimmune rheumatic diseases: a narrative review. Acta Clin Belg (2021) 19:1–9. doi: 10.1080/17843286.2021.1992582 34666637

[8] Zhao CN Mei YJ Wu GC Mao YM Wu Q Dan YL. Effect of air pollution on hospital admissions for systemic lupus erythematosus in bengbu, China: a time series study. Lupus (2019) 28(13):1541–8. doi: 10.1177/0961203319882503 31615325

[9] He YS Wang GH Wu Q Wu ZD Chen Y Tao JH. The relationship between ambient air pollution and hospitalizations for gout in a humid subtropical region of China. J Inflammation Res (2021) 14:5827–35. doi: 10.2147/JIR.S329706 PMC8575452 34764674

[10] Oh I Lee J Ahn K Kim J Kim YM Sun Sim C. Association between particulate matter concentration and symptoms of atopic dermatitis in children living in an industrial urban area of south Korea. Environ Res (2018) 160:462–8. doi: 10.1016/j.envres.2017.10.030 29078139

[11] Li X Zhou LX Yang LL Huang XL Wang N Hu YG. The relationship between short-term PM2.5 exposure and outpatient visits for acne vulgaris in chongqing, China: a time-series study. Environ Sci pollut Res Int (2022) 29(40):61502–11. doi: 10.1007/s11356-022-20236-8 35442002

[12] Kim HJ Bae IH Son ED Park J Cha N Na HW. Transcriptome analysis of airborne PM2.5-induced detrimental effects on human keratinocytes. Toxicol Lett (2017) 273:26–35. doi: 10.1016/j.toxlet.2017.03.010 28341207

[13] Feng S Gao D Liao F Zhou F Wang X. The health effects of ambient PM2.5 and potential mechanisms. Ecotoxicol Environ Saf (2016) 128:67–74. doi: 10.1016/j.ecoenv.2016.01.030 26896893

[14] Latzin P Frey U Armann J Kieninger E Fuchs O Röösli M. Exposure to moderate air pollution during late pregnancy and cord blood cytokine secretion in healthy neonates. PloS One (2011) 6(8):e23130. doi: 10.1371/journal.pone.0023130 21826232 PMC3149643

[15] Zhao R Zhou H Su SB. A critical role for interleukin-1β in the progression of autoimmune diseases. Int Immunopharmacol (2013) 17(3):658–69. doi: 10.1016/j.intimp.2013.08.012 24012439

[16] Galperín G Berra M Marquez MI Mandaradoni M Tau J Berra A. Impact of environmental pollution on the ocular surface of sjögren's syndrome patients. Arq Bras Oftalmol (2018) 81(6):481–9. doi: 10.5935/0004-2749.20180091 30231159

[17] Bernatsky S Smargiassi A Johnson M Kaplan GG Barnabe C Svenson L. Fine particulate air pollution, nitrogen dioxide, and systemic autoimmune rheumatic disease in Calgary, Alberta. Environ Res (2015) 140:474–8. doi: 10.1016/j.envres.2015.05.007 PMC4492844 25988990

[18] Bai YC Wang CY Lin CL Lai JN Wei JC. Association between air pollution and the risk of uveitis: A nationwide, population-based cohort study. Front Immunol (2021) 12:613893. doi: 10.3389/fimmu.2021.613893 33815370 PMC8013994

[19] Wu Q Xu Z Dan YL Cheng J Zhao CN Mao YM. Association between traffic-related air pollution and hospital readmissions for rheumatoid arthritis in hefei, China: A time-series study. Environ pollut (2021) 268(Pt A):115628. doi: 10.1016/j.envpol.2020.115628 33049484

[20] Chen Y He YS Feng YT Wu ZD Wang J Yin KJ. The effect of air pollution exposure on risk of outpatient visits for sjogren's syndrome: A time-series study. Environ Res (2022) 214:114017. doi: 10.1016/j.envres.2022.114017 35981608

[21] Zhao CN Xu Z Wu GC Mao YM Liu LN Qian-Wu. Emerging role of air pollution in autoimmune diseases. Autoimmun Rev (2019) 18(6):607–14. doi: 10.1016/j.autrev.2018.12.010 30959217

[22] Fu H Liu X Li W Zu Y Zhou F Shou Q. PM2.5 exposure induces inflammatory response in macrophages *via* the TLR4/COX-2/NF-κB pathway. Inflammation (2020) 43(5):1948–58. doi: 10.1007/s10753-020-01269-y 32504162

[23] Veldhoen M Hirota K Westendorf AM Buer J Dumoutier L Renauld JC. The aryl hydrocarbon receptor links TH17-cell-mediated autoimmunity to environmental toxins. Nature (2008) 453(7191):106–9. doi: 10.1038/nature06881 18362914

[24] Quintana FJ Basso AS Iglesias AH Korn T Farez MF Bettelli E. Control of t(reg) and T(H)17 cell differentiation by the aryl hydrocarbon receptor. Nature (2008) 453(7191):65–71. doi: 10.1038/nature06880 18362915

[25] van Voorhis M Knopp S Julliard W Fechner JH Zhang X Schauer JJ. Exposure to atmospheric particulate matter enhances Th17 polarization through the aryl hydrocarbon receptor. PloS One (2013) 8(12):e82545. doi: 10.1371/journal.pone.0082545 24349309 PMC3859609

[26] Xin L Zhu Y Liu J Fang Y Xie J. Exposure-lag-response associations between extreme environmental conditions and primary sjögren's syndrome. Clin Rheumatol (2022) 41(2):523–32. doi: 10.1007/s10067-021-05910-5 34523037

[27] Aulock SV Deininger S Draing C Gueinzius K Dehus O Hermann C. Gender difference in cytokine secretion on immune stimulation with LPS and LTA. J Interferon Cytokine Res (2006) 26(12):887–92. doi: 10.1089/jir.2006.26.887 17238831

[28] Simon AK Hollander GA McMichael A. Evolution of the immune system in humans from infancy to old age. Proc Biol Sci (2015) 282(1821):20143085. doi: 10.1098/rspb.2014.3085 26702035 PMC4707740

[29] López-Miguel A Tesón M Martín-Montañez V Enríquez-de-Salamanca A Stern ME González-García MJ. Clinical and molecular inflammatory response in sjögren syndrome-associated dry eye patients under desiccating stress. Am J Ophthalmol (2016) 161:133–41. doi: 10.1016/j.ajo.2015.09.039 26456254

