# Supplementary material for: Exposure to particulate pollutant increases the risk of hospitalizations for Sjögren’s syndrome
Source: Front Immunol. 2022 Dec 15;13:1059981. doi: 10.3389/fimmu.2022.1059981 (PMC9798840; doi:10.3389/fimmu.2022.1059981)
Supplement: Supplementary file 1 [file DataSheet_1.doc]

**Table S1** The association between PM_2.5_ exposure and SS hospitalizations in Hefei from 2016 to 2021

| Single-day | *RR* (95% *CI*) | Multi-day | *RR* (95% *CI*) |
| --- | --- | --- | --- |
| 0 | 0.980(0.954-1.007) | 0-0 | 0.980(0.954-1.007) |
| 1 | 0.998(0.984-1.012) | 0-1 | 0.978(0.940-1.018) |
| 2 | 1.011(0.999-1.024) | 0-2 | 0.989(0.948-1.033) |
| 3 | 1.015(1.001-1.029) ^a^ | 0-3 | 1.004(0.959-1.051) |
| 4 | 1.009(0.998-1.021) | 0-4 | 1.014(0.966-1.064) |
| 5 | 0.998(0.986-1.010) | 0-5 | 1.011(0.961-1.065) |
| 6 | 0.983(0.961-1.005) | 0-6 | 0.994(0.938-1.053) |

RR: Relative risk; CI: confidence interval; a:*P* < 0.05.

**Table S2** The association between PM_2.5_ exposure and SS hospitalizations in different subgroups (*RR*, 95% *CI*)

| lag | Female | Age ≤ 40 years | 41 years ≤ Age ≤ 64 years | Age ≥ 65 years | Hot season | Cold season |
| --- | --- | --- | --- | --- | --- | --- |
| 0 | 0.982(0.954-1.011) | 0.968(0.895-1.045) | 0.991(0.954-1.029) | 0.976(0.925(1.029) | 0.987(0.937-1.039) | 0.980(0.957-1.004) |
| 1 | 1.001(0.988-1.015) | 0.990(0.954-1.027) | 1.002(0.985-1.021) | 1.004(0.979-1.030) | 1.001(0.977-1.026) | 1.000(0.989-1.012) |
| 2 | 1.013(1.000-1.027) ^a^ | 1.005(0.971-1.040) | 1.010(0.993-1.027) | 1.023(1.000-1.047) ^a^ | 1.008(0.985-1.032) | 1.013(1.002-1.024) ^a^ |
| 3 | 1.015(1.001-1.029) ^a^ | 1.009(0.974-1.046) | 1.012(0.995-1.030) | 1.029(1.004-1.054) ^a^ | 1.006(0.981-1.032) | 1.016(1.005-1.028) ^a^ |
| 4 | 1.009(0.998-1.020) | 1.005(0.978-1.033) | 1.010(0.996-1.024) | 1.024(1.004-1.044) ^a^ | 0.996(0.975-1.017) | 1.011(1.002-1.020) ^a^ |
| 5 | 0.997(0.985-1.009) | 0.996(0.967-1.025) | 1.004(0.990-1.020) | 1.012(0.991-1.035) | 0.981(0.960-1.003) | 1.001(0.991-1.011) |
| 6 | 0.983(0.961-1.005) | 0.984(0.931-1.040) | 0.998(0.971-1.026) | 0.998(0.959-1.038) | 0.964(0.928-1.001) | 0.989(0.971-1.007) |
| 0-0 | 0.982(0.954-1.011) | 0.968(0.895-1.045) | 0.991(0.954-1.029) | 0.976(0.925-1.029) | 0.987(0.937-1.039) | 0.980(0.957-1.004) |
| 0-1 | 0.983(0.944-1.024) | 0.958(0.859-1.068) | 0.993(0.942-1.048) | 0.980(0.909-1.056） | 0.988(0.918-1.062) | 0.981(0.948-1.015) |
| 0-2 | 0.996(0.954-1.041) | 0.963(0.857-1.082) | 1.004(0.948-1.063) | 1.003(0.925-1.087) | 0.996(0.921-1.077) | 0.994(0.958-1.031) |
| 0-3 | 1.011(0.965-1.059) | 0.972(0.860-1.098) | 1.016(0.956-1.079) | 1.031(0.947-1.122) | 1.002(0.923-1.088) | 1.010(0.971-1.050) |
| 0-4 | 1.020(0.971-1.072) | 0.977(0.860-1.110) | 1.026(0.962-1.094) | 1.056(0.965-1.155) | 0.998(0.913-1.090) | 1.021(0.979-1.064) |
| 0-5 | 1.017(0.965-1.071) | 0.972(0.852-1.109) | 1.030(0.964-1.102) | 1.069(0.972-1.176) | 0.979(0.890-1.076) | 1.022(0.978-1.068) |
| 0-6 | 0.999(0.942-1.060) | 0.956(0.826-1.107) | 1.028(0.954-1.108) | 1.066(0.956-1.189) | 0.943(0.847-1.051) | 1.011(0.962-1.063) |

*RR:* Relative risk; *CI:* confidence interval; a:*P* < 0.05.

**Table S3** The association between PM_10_ exposure and SS hospitalizations in Hefei from 2016 to 2021

| Single-day | *RR* (95% *CI*) | Multi-day | *RR* (95% *CI*) |
| --- | --- | --- | --- |
| 0 | 1.005(0.975-1.036) | 0-0 | 1.005(0.975-1.036) |
| 1 | 0.995(0.978-1.014) | 0-1 | 1.000(0.969-1.033) |
| 2 | 1.005(0.993-1.017) | 0-2 | 1.005(0.972-1.039) |
| 3 | 1.013(1.001-1.026) ^a^ | 0-3 | 1.019(0.985-1.054) |
| 4 | 1.011(1.000-1.026) ^a^ | 0-4 | 1.030(0.992-1.068) |
| 5 | 1.000(0.992-1.009) | 0-5 | 1.030(0.991-1.070) |
| 6 | 0.986(0.969-1.004) | 0-6 | 1.016(0.976-1.058) |

*RR*: Relative risk; *CI*: confidence interval; a:*P* < 0.05.

**Table S4** The association between PM_10_ exposure and SS hospitalizations in different subgroups (*RR*, 95% *CI*)

| lag | Female | Age ≤ 40 years | 41 years ≤ Age ≤ 64 years | Age ≥ 65 years | Hot season | Cold season |
| --- | --- | --- | --- | --- | --- | --- |
| 0 | 1.005(0.975-1.037) | 0.982(0.926-1.042) | 0.993(0.953-1.034) | 1.014(0.963-1.066) | 1.007(0.966-1.051) | 0.997(0.971-1.025) |
| 1 | 0.997(0.979-1.015) | 1.000(0.974-1.028) | 1.000(0.978-1.024) | 0.999(0.969-1.029) | 0.987(0.965-1.010) | 0.999(0.982-1.015) |
| 2 | 1.006(0.994-1.019) | 1.013(0.990-1.037) | 1.010(0.994-1.026) | 1.011(0.991-1.032) | 0.997(0.981-1.012) | 1.008(0.997-1.020) |
| 3 | 1.014(1.002-1.027) ^a^ | 1.019(0.994-1.045) | 1.015(1.001-1.030) ^a^ | 1.024(1.003-1.044) ^a^ | 1.007(0.993-1.022) | 1.015(1.004-1.026) ^a^ |
| 4 | 1.020(0.999-1.022) | 1.019(1.001-1.039) ^a^ | 1.013(0.999-1.028) | 1.023(1.004-1.043) ^a^ | 1.004(0.990-1.019) | 1.012(1.002-1.023) ^a^ |
| 5 | 0.999(0.990-1.008) | 1.016(0.996-1.037) | 1.007(0.997-1.018) | 1.014(1.000-1.029) ^a^ | 0.991(0.980-1.003) | 1.004(0.996-1.012) |
| 6 | 0.984(0.966-1.002) | 1.011(0.974-1.050) | 0.999(0.977-1.022) | 1.001(0.971-1.031) | 0.974(0.952-0.996) | 0.993(0.976-1.009) |
| 0-0 | 1.005(0.975-1.037) | 0.982(0.926-1.042) | 0.993(0.953-1.034) | 1.014(0.963-1.066) | 1.007(0.966-1.051) | 0.997(0.971-1.025) |
| 0-1 | 1.002(0.970-1.035) | 0.983(0.905-1.067) | 0.993(0.951-1.037) | 1.012(0.959-1.068) | 0.994(0.949-1.042) | 0.996(0.968-1.025) |
| 0-2 | 1.009(0.975-1.043) | 0.996(0.913-1.086) | 1.003(0.959-1.049) | 1.023(0.967-1.082) | 0.991(0.944-1.040) | 1.005(0.975-1.035) |
| 0-3 | 1.023(0.988-1.059) | 1.015(0.929-1.108) | 1.018(0.972-1.066) | 1.047(0.989-1.109) | 0.998(0.949-1.050) | 1.019(0.988-1.052) |
| 0-4 | 1.033(0.995-1.073) | 1.034(0.944-1.133) | 1.032(0.981-1.085) | 1.072(1.007-1.140) ^a^ | 1.002(0.950-1.058) | 1.032(0.998-1.068) |
| 0-5 | 1.033(0.993-1.074) | 1.051(0.957-1.154) | 1.039(0.986-1.095) | 1.087(1.018-1.160) ^a^ | 0.994(0.938-1.052) | 1.036(1.000-1.073) ^a^ |
| 0-6 | 1.016(0.975-1.059) | 1.063(0.958-1.789) | 1.038(0.983-1.097) | 1.087(1.015-1.165) ^a^ | 0.968(0.909-1.030) | 1.029(0.992-1.067) |

*RR:* Relative risk; *CI:* confidence interval; a:*P* < 0.05.


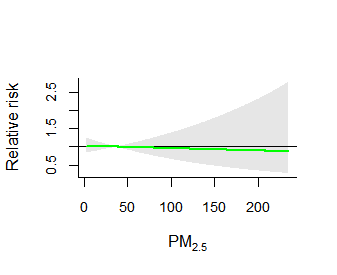

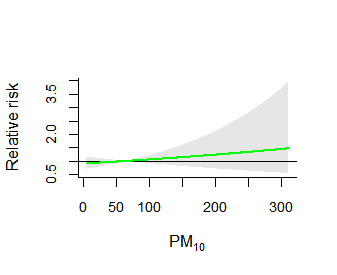


**Figure S1** Concentration-response curves of daily hospitalizations for SS and PM_2.5_, PM_10_ levels


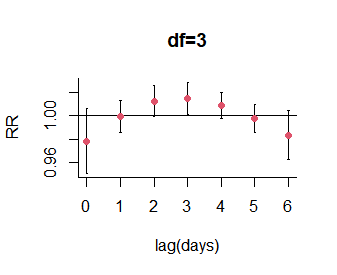

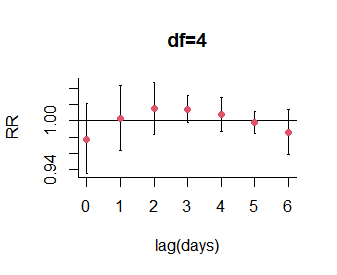

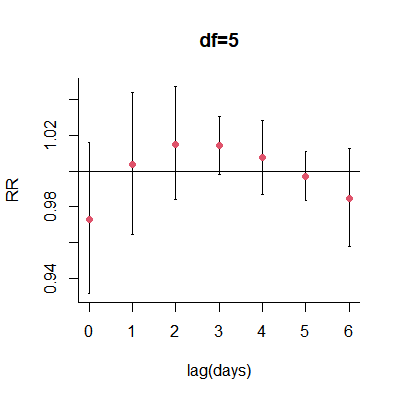

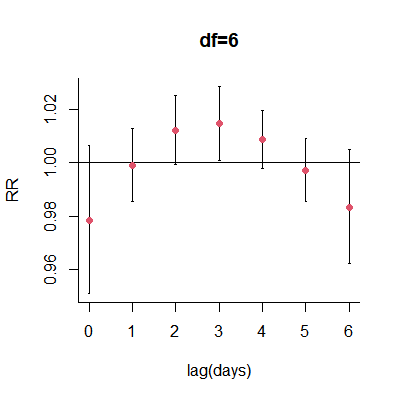

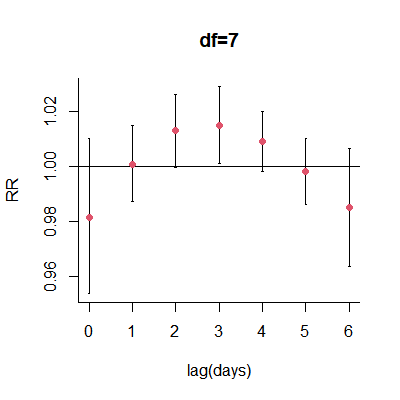

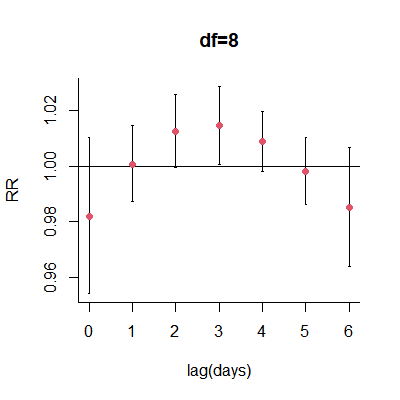


When the dfs for time is 6, we adjust the dfs for air pollution from 3 to 5 in turn, and

**Figure S2** The single-day association between PM_2.5_ exposure and SS hospitalizations

when varying the degrees of freedom (3-5*dfs*) for NO_2_, MT, RH and

the df (6-8 *dfs*/year) for time


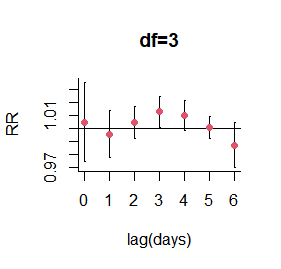

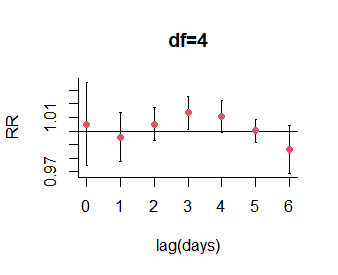

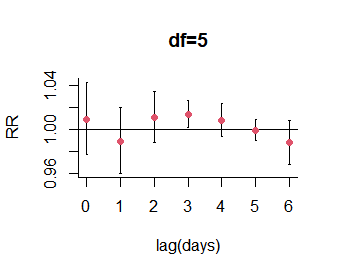

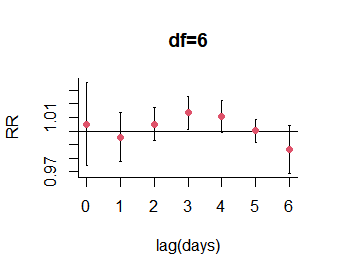

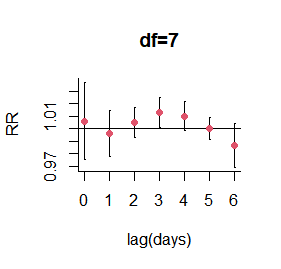

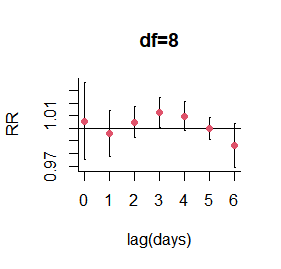


**Figure S3** The single-day association between PM_10_ exposure and SS hospitalizations

when varying the degrees of freedom (3-5*dfs*) for NO_2_, MT, RH and

the df (6-8 *dfs*/year) for time
